# Construction and validation of a prognostic model for clinical outcomes in patients with prolonged disorders of consciousness based on multidimensional indicators: a prospective cohort study

**DOI:** 10.3389/fneur.2026.1738430

**Published:** 2026-02-20

**Authors:** Zhen Feng, Qiaojun Zhang

**Affiliations:** The Second Affiliated Hospital of Xi’an Jiaotong University (Xibei Hospital), Xi’an, Shanxi, China

**Keywords:** behavioral scales, clinical evaluation, disorders of consciousness, prediction model, prognosis

## Abstract

**Objective:**

To develop and validate a prognostic prediction model for patients with prolonged disorders of consciousness (pDoC) based on multidimensional clinical indicators, aiming to improve prognostic accuracy and provide objective support for clinical decision-making.

**Methods:**

A prospective cohort study was conducted involving 304 patients with pDoC admitted to the First Affiliated Hospital of Nanchang University between January 2021 and October 2023. Clinical data were collected, including demographics, etiology, disease duration, behavioral assessment scores (Glasgow Coma Scale [GCS], Full Outline of UnResponsiveness [FOUR], and Coma Recovery Scale-Revised [CRS-R]), laboratory indicators, and other relevant clinical variables. Prognosis was assessed using the Glasgow Outcome Scale-Extended (GOS-E) and dichotomized into good outcome (GOS-E 3–8) and poor outcome (GOS-E 1–2). Feature selection was performed using the Boruta algorithm combined with recursive feature elimination (RFE). Prognostic models were developed using logistic regression, support vector machine (SVM), multilayer perceptron (MLP), XGBoost, and gradient boosting machine (GBM) implemented in scikit-learn. Model performance was evaluated using accuracy, receiver operating characteristic curve area under the curve (ROC-AUC), and decision curve analysis (DCA). SHapley Additive exPlanations (SHAP) were applied to interpret the optimal model.

**Results:**

Among the developed models, the GBM model demonstrated the best predictive performance, with an AUROC of 0.954 (95% CI: 0.924–0.977) in the training set and 0.922 (95% CI: 0.847–0.979) in the test set. Decision curve analysis indicated that the GBM model yielded substantial net clinical benefit across most threshold probabilities. SHAP analysis identified CRS-R score, age, FOUR score, total GCS score, and length of hospitalization as the most influential prognostic predictors.

**Conclusion:**

A robust prognostic prediction model for pDoC patients was developed and validated using multidimensional clinical data and machine learning techniques. The GBM model achieved excellent discriminative performance and clinical utility, providing an objective tool for prognosis estimation and individualized treatment and rehabilitation planning. Further multi-center studies are warranted to optimize the model and confirm its generalizability.

## 1 Introduction

Disorders of Consciousness (DoC) are caused by brain injuries resulting from traumatic brain injury (TBI), stroke, hemorrhage, hypoxia, or other factors that disrupt overall brain function, leading to a loss of consciousness. This includes conditions such as coma, Unresponsive Wakefulness Syndrome (UWS), and Minimally Conscious State (MCS) (1). Prolonged Disorders of Consciousness (pDoC) (2) is a more complex and severe form of DoC. A patient is diagnosed with pDoC if they remain in an unconscious state, unresponsive to both themselves and the environment, for more than 28 days. With continuous advancements in emergency medicine and critical care, the survival rate of patients with severe brain injuries has significantly increased, resulting in a growing number of individuals living with chronic consciousness impairment (3). Epidemiological studies indicate that the number of pDoC patients in China currently exceeds 500,000, with an annual increase of over 100,000 cases and cumulative direct medical expenses surpassing 10 billion RMB (4); Further research by Leonard et al. revealed that the annual medical costs for pDoC patients are approximately 120,000 euros, while the median annual income of 40.2% of family caregivers is around 170,000 euros (5). This not only causes immense suffering for the patients themselves but also places a long-term caregiving burden and psychological stress on their family members, creating significant challenges for the healthcare system (6).

Accurate prognostic assessments provide essential information for patients and their families, helping family members create long-term care plans, allocate resources effectively, and manage psychological expectations, thereby alleviating the burden and stress associated with prolonged caregiving. Additionally, precise prognosis prediction aids healthcare providers in designing more targeted treatment and rehabilitation strategies, optimizing the allocation of medical resources, and preventing both overtreatment and undertreatment, ultimately improving the efficiency and effectiveness of the healthcare system. This approach is crucial for addressing the increasing number of pDoC patients. Therefore, accurate prognosis prediction is key to developing appropriate rehabilitation plans, optimizing resource allocation, and offering psychological support and expectation management for the families of patients (7).

Currently, the prognostic evaluation of pDoC patients primarily relies on a combination of behavioral assessment scales, neurophysiological examinations, neuroimaging technologies, and serum biomarkers. Behavioral assessment scales are widely used in clinical practice due to their simplicity and ease of administration. These include the Glasgow Coma Scale (GCS), the Full Outline of Unresponsiveness (FOUR) score, and the Coma Recovery Scale-Revised (CRS-R), among others. Since its introduction in 1974, the GCS has become the most commonly used tool for assessing consciousness (8), though it has limitations such as a limited number of assessment items and an inability to evaluate brainstem reflexes (9). The FOUR score overcomes some of these limitations by incorporating assessments of brainstem reflexes and respiratory patterns (10). The CRS-R is considered the gold standard for diagnosing and assessing pDoC (11), as it effectively distinguishes between patients in UWS and MCS and is sensitive to subtle behavioral changes. However, its comprehensive assessment is time-consuming and requires extensive training for evaluators (12). Despite their usefulness, The CRS-R is influenced by factors such as fluctuations in patient consciousness, medication effects, complications, and the subjective judgments of the evaluator, leading to a misdiagnosis rate as high as 40% (13). Furthermore, these scales rely mainly on the patient’s overt behavioral responses and are unable to assess patients with severe motor dysfunction who may retain cognitive abilities (14).

Neuroimaging and neurophysiological techniques provide essential structural and functional information, offering valuable prognostic data for the assessment of pDoC patients (15). However, their high costs, limited availability, and dependence on specialized personnel hinder their widespread application in routine clinical practice (16, 17). Serum biomarkers hold significant potential for prognostic prediction in consciousness disorders (18), but their clinical utility is limited due to a lack of sufficient research, with relatively few studies conducted specifically in pDoC patients. Research by Bagnato et al. has demonstrated that serum biomarkers can not only more accurately represent the extent of brain damage but also support prediction models with valuable data (19).

In recent years, with the rapid advancement of artificial intelligence technology, machine learning-based prediction models have offered new solutions to the challenges mentioned above. Compared to traditional statistical methods, machine learning techniques present several advantages, such as the ability to handle high-dimensional data, capture complex nonlinear relationships between variables, automatically assess the importance of each predictive factor, possess dynamic learning capabilities, and provide individualized predictions. Machine learning has already shown significant potential in the field of pDoC prognostic prediction (20). Haveman et al. defined a Glasgow Outcome Scale-Extended (GOSE) score ≥3 as the threshold for good prognosis in pDoC patients (21). They developed a weighted sparse brain network feature based on resting-state functional magnetic resonance imaging (fMRI) data and utilized a support vector machine classifier to predict patient outcomes, achieving a classification accuracy of 90% (22). While machine learning has demonstrated substantial potential in pDoC prognostic prediction, its clinical application still faces challenges, such as data quality issues, lack of model interpretability, limited generalization capacity, and class imbalance (23–25).

Building on the above analysis, this study integrates multidimensional indicators, such as behavioral assessments and serum biomarkers, and applies advanced machine learning algorithms to construct a multimodal prediction model for prognostic evaluation in pDoC patients.

## 2 Materials and methods

### 2.1 Study subjects

This study is a prospective cohort study that included 304 patients with DoC who were treated in the Department of Rehabilitation Medicine at the First Affiliated Hospital of Nanchang University from January 2021 to October 2023.

Inclusion criteria: ① First diagnosis of DoC; ② UWS or MCS according to the “European Guidelines for Coma and Consciousness Disorder Diagnosis; ③ Duration of DoC ≥28 days.

Exclusion criteria: ① Co-existing severe brain atrophy, congenital neurological disorders, or drug poisoning; ② History of cognitive dysfunction (such as dementia or a history of mental illness).

### 2.2 Research methods

#### 2.2.1 Data collection

Clinical data were collected for all patients at the time of hospital admission as part of the baseline assessment. The data collected included demographic characteristics (age, gender, education level, occupational status, marital status), etiology, duration of DoC, length of hospitalization(defined as the total duration of inpatient stay during the rehabilitation phase), smoking history, behavioral assessment scales (GCS score, FOUR score, CRS-R score), medical history (hypertension, diabetes, history of multiple injuries, history of craniectomy), pupil dilation (defined as a diameter ≥5 mm, unilateral, bilateral, or none), complications (secondary epilepsy, muscle spasticity, pulmonary infections), imaging findings (subdural effusion, subarachnoid hemorrhage, hydrocephalus), and laboratory test indicators (albumin, hemoglobin, etc.). These variables were all obtained as part of the routine clinical evaluation at admission.

#### 2.2.2 Outcome measures

The GOS-E score was used as the outcome variable. This score was assessed by two follow-up personnel who were rigorously and professionally trained. Follow-up was conducted through telephone calls to the patients’ families, with a follow-up interval of no more than 2 weeks. If at least 3 phone contact attempts were unsuccessful, the patient was considered lost to follow-up. The GOS-E scale consists of 8 categories (26), which include: ① Death; ② Vegetative state (unconscious state); ③ Severe disability (conscious but requiring frequent assistance for daily activities); ④ Moderate to severe disability (conscious but requiring partial assistance for daily activities); ⑤ Moderate disability (independent in daily life but unable to participate in some social roles); ⑥ Mild disability (able to participate in social roles but with limitations); ⑦ Good recovery (return to normal life but with minor symptoms); ⑧ Complete recovery (full recovery to normal life without functional impairments). IIn this study, the patients’ outcomes were categorized based on the GOS-E score into two groups: a poor prognosis group (GOS-E score 1–2) and a good prognosis group (GOS-E score 3–8) (21). Outcome assessment was performed 1 year after the initial baseline data collection at hospital admission, providing a consistent and clinically relevant evaluation window for assessing long-term recovery in patients with DoC. This standardized assessment time point improves the reproducibility and clinical applicability of the model.

### 2.3 Data preprocessing

In this study, we conducted rigorous screening and preprocessing of the data from patients with DoC who were treated at the Department of Rehabilitation Medicine, The First Affiliated Hospital of Nanchang University, between January 2021 and October 2023. The initial cohort included 304 patients, with cases exhibiting missing critical data being excluded. Missing critical data included essential assessment scales, laboratory results, or follow-up data. Ultimately, data from 239 patients with good completeness were included in the final analysis. The data set was divided into a training set (70%) and a testing set (30%) using stratified random sampling, ensuring that the proportion of samples in each category was consistent between the two sets to enhance the model’s generalizability.

### 2.4 Feature selection

This study employed a combination of the Boruta algorithm based on random forests and recursive feature elimination (RFE) for feature selection. The Boruta algorithm constructs random features as a control group and compares their importance with that of the original features, thereby identifying those features that are truly important for the target variable. Recursive feature elimination utilizes feature contribution analysis and model average AUC performance evaluation to quantify the contribution of features to the model output and assess the model’s classification performance, further validating the importance of the features. The combination of these two methods ensures the comprehensiveness and robustness of the feature selection process, while enhancing the predictive power of the feature subset.

### 2.5 Machine learning algorithms

This study constructed five machine learning models using Python’s scikit-learn library: Logistic Regression (LR), Support Vector Machine (SVM), XGBoost, Gradient Boosting Machine (GBM), and Multi-Layer Perceptron (MLP). The optimal parameters for each model were determined using Grid Search and 5-fold cross-validation (CV) to minimize overfitting and optimize model performance. Logistic Regression (LR), a classic statistical method, is simple yet performs exceptionally well in medical predictions, offering good interpretability. Support Vector Machine (SVM) constructs a linear decision boundary by mapping input vectors into a high-dimensional feature space, thereby enhancing generalization ability. Multi-Layer Perceptron (MLP), a feedforward neural network model, possesses powerful representation learning capabilities. XGBoost is one of the most powerful boosting algorithms, offering excellent predictive performance via gradient boosting frameworks and regularization techniques. Gradient Boosting Machine (GBM) sequentially integrates multiple weak learners to improve overall performance.

In addition to conventional discrimination metrics such as the area under the receiver operating characteristic curve (AUC), the Net Reclassification Improvement (NRI) was employed as a supplementary performance measure. NRI, originally proposed by Pencina MJ et al., evaluates whether a new prediction model improves individual risk stratification relative to a reference model by quantifying changes in risk category assignment for individuals with and without events (27). Unlike AUC, which summarizes overall discriminative ability, NRI specifically assesses the direction and magnitude of risk reclassification, providing complementary information on model performance.

In this study, the machine learning model demonstrating the best performance based on cross-validation was treated as the new model and was compared with LR, which served as the reference model. The NRI was calculated using predefined clinical risk thresholds to assess differences in patient risk reclassification between the two models.

### 2.6 SHAP interpretability analysis

To enhance clinical applicability and interpretability, SHAP analysis was employed to evaluate the contribution of each feature to the model’s predictions. SHAP, based on the Shapley values from game theory, quantifies each feature’s contribution to the model’s prediction, with theoretical guarantees of completeness, consistency, and local accuracy. By calculating each feature’s marginal contribution across all possible feature combinations, SHAp values reflect the positive or negative influence of features on predictions. In this study, SHAP values were computed for all samples, and features were ranked based on mean absolute SHAP values. SHAP dependence plots were used to explore nonlinear relationships between feature values and their effects. Additionally, SHAP force plots visually demonstrate how features collectively influence individual sample predictions, providing explanations for personalized prognostic predictions.

### 2.7 Statistical analysis

All data analysis was conducted using Python 3.8 and related libraries. Continuous variables were expressed as mean ± standard deviation or median (interquartile range), while categorical variables were presented as frequencies and percentages. Group comparisons were performed using independent sample t-tests, Mann–Whitney U tests, or chi-square tests, with the appropriate statistical method selected based on data distribution characteristics. For model evaluation, the area under the receiver operating characteristic curve (AUC) was used to assess the classification performance of each model. DCA was used to compare the clinical utility of each model, and calibration curves were employed to assess the consistency between predicted probabilities and actual outcomes. Statistical analyses were carried out using Python libraries, including scikit-learn (version 1.5.1) and statsmodels (version 0.14.4), with visualization generated by matplotlib (version 3.10.0). Data preprocessing relied on pandas (version 2.2.3) and numpy (version 1.26.4), model construction used xgboost (version 2.0.3), and model interpretation was facilitated using shap (version 0.45.1). All statistical tests were two-sided, and a *p*-value of < 0.05 was considered statistically significant.

## 3 Results

### 3.1 Patient characteristics

A total of 304 patients with Disorders of Consciousness (DoC) who visited the Department of Rehabilitation Medicine at The First Affiliated Hospital of Nanchang University from January 2021 to October 2023 were initially included. After strict screening and excluding cases with missing data, 239 patients were ultimately included in the analysis. Among them, 97 patients had a good prognosis (GOS-E score 3–8), while 142 had a poor prognosis (GOS-E score 1–2). The mean age of the entire cohort was 53.7 ± 15.0 years, with 60.7% of the patients being male. As shown in Table 1, there were statistically significant differences between the good and poor prognosis groups in terms of demographic characteristics, consciousness status, and assessment indicators (*p* < 0.05). No substantial differences were observed between the training and validation sets. Table 2 reveals that there were no significant differences between the training and validation sets in terms of demographic characteristics, medical history, consciousness status, and physiological indicators, except for the presence of subdural effusion, which showed a statistically significant difference (*p* < 0.05).

**Table 1:** Basic clinical information of the good prognosis and poor prognosis groups.

Variables	Total (*n* = 239)	Poor prognosis group (*n* = 142)	Good prognosis group (*n* = 97)	*p* value
History_of_hypertension				
No	147 (61.5%)	83 (58.5%)	64 (66.0%)	
Yes	92 (38.5%)	59 (41.5%)	33 (34.0%)	0.299
History_of_diabetes_mellitus				
No	211 (88.3%)	124 (87.3%)	87 (89.7%)	
Yes	28 (11.7%)	18 (12.7%)	10 (10.3%)	0.723
History_of_epilepsy_disorder				
No	228 (95.4%)	133 (93.7%)	95 (97.9%)	
Yes	11 (4.6%)	9 (6.3%)	2 (2.1%)	0.217
Pulmonary_infection				
No	72 (30.1%)	41 (28.9%)	31 (32.0%)	
Yes	167 (69.9%)	101 (71.1%)	66 (68.0%)	0.714
Subdural_effusion				
No	204 (85.4%)	129 (90.8%)	75 (77.3%)	
Yes	35 (14.6%)	13 (9.2%)	22 (22.7%)	0.007
Smoking_history				
No	226 (94.6%)	132 (93.0%)	94 (96.9%)	
Yes	13 (5.4%)	10 (7.0%)	3 (3.1%)	0.302
Sex				
Female	94 (39.3%)	62 (43.7%)	32 (33.0%)	
Male	145 (60.7%)	80 (56.3%)	65 (67.0%)	0.128
Multiple_injuries				
No	180 (75.3%)	108 (76.1%)	72 (74.2%)	
Yes	59 (24.7%)	34 (23.9%)	25 (25.8%)	0.866
Craniotomy				
No	171 (71.5%)	104 (73.2%)	67 (69.1%)	
Yes	68 (28.5%)	38 (26.8%)	30 (30.9%)	0.579
Subarachnoid_hemorrhage				
No	169 (70.7%)	103 (72.5%)	66 (68.0%)	
Yes	70 (29.3%)	39 (27.5%)	31 (32.0%)	0.545
Hydrocephalus				
No	206 (86.2%)	122 (85.9%)	84 (86.6%)	
Yes	33 (13.8%)	20 (14.1%)	13 (13.4%)	1.000
Muscle_tone				
Unable to measure	78 (32.6%)	48 (33.8%)	30 (30.9%)	
Weak	37 (15.5%)	23 (16.2%)	14 (14.4%)	
Normal	77 (32.2%)	40 (28.2%)	37 (38.1%)	
High	47 (19.7%)	31 (21.8%)	16 (16.5%)	0.411
Etiology				
Traumatic brain injury	95 (39.7%)	57 (40.1%)	38 (39.2%)	
Intracerebral hemorrhage	117 (49.0%)	66 (46.5%)	51 (52.6%)	
Cerebral infarction	7 (2.9%)	3 (2.1%)	4 (4.1%)	
Others	20 (8.4%)	16 (11.3%)	4 (4.1%)	0.190
Patient’s_state_of_consciousness				
VS	150 (62.8%)	111 (78.2%)	39 (40.2%)	
MCS-	68 (28.5%)	26 (18.3%)	42 (43.3%)	
MCS+	16 (6.7%)	5 (3.5%)	11 (11.3%)	
Emergence from MCS	5 (2.1%)	0 (0.0%)	5 (5.2%)	0.000
Age				
Mean ± SD	53.7 ± 15.0	56.8 ± 14.2	49.0 ± 15.2	0.000
Hb				
Mean ± SD	101.5 ± 15.7	103.1 ± 14.9	99.3 ± 16.5	0.075
D_dimer				
Mean ± SD	3.2 ± 4.5	3.3 ± 4.1	3.1 ± 5.1	0.760
Serum_albumin				
Mean ± SD	35.8 ± 6.9	35.3 ± 4.9	36.6 ± 9.1	0.207
Length_of_hospital_stay				
Mean ± SD	31.4 ± 14.1	30.4 ± 15.9	32.8 ± 10.8	0.150
CRS-R				
Mean ± SD	7.3 ± 3.2	6.1 ± 2.3	9.1 ± 3.5	0.000
GCS				
Mean ± SD	8.2 ± 1.8	7.6 ± 1.6	9.2 ± 1.5	0.000
FOUR				
Mean ± SD	12.0 ± 2.2	11.2 ± 2.0	13.2 ± 1.8	0.000

Continuous variables are presented as mean ± standard deviation and were compared between groups using the independent-samples *t*-test. Categorical variables are presented as counts (percentages) and were compared using the Chi-square test or Fisher’s exact test, as appropriate. A *p* value < 0.05 was considered statistically significant.

**Table 2:** Clinical information of the training and validation sets.

Variables	Total (*n* = 239)	Training set (*n* = 167)	Validation set (*n* = 72)	*p* value
History_of_hypertension				
No	147 (61.5%)	102 (61.1%)	45 (62.5%)	
Yes	92 (38.5%)	65 (38.9%)	27 (37.5%)	0.950
History_of_diabetes_mellitus				
No	211 (88.3%)	147 (88.0%)	64 (88.9%)	
Yes	28 (11.7%)	20 (12.0%)	8 (11.1%)	1.000
History_of_epilepsy_disorder				
No	228 (95.4%)	159 (95.2%)	69 (95.8%)	
Yes	11 (4.6%)	8 (4.8%)	3 (4.2%)	1.000
Pulmonary_infection				
No	72 (30.1%)	52 (31.1%)	20 (27.8%)	
Yes	167 (69.9%)	115 (68.9%)	52 (72.2%)	0.715
Subdural_effusion				
No	204 (85.4%)	141 (84.4%)	63 (87.5%)	
Yes	35 (14.6%)	26 (15.6%)	9 (12.5%)	0.677
Smoking_history				
No	226 (94.6%)	158 (94.6%)	68 (94.4%)	
Yes	13 (5.4%)	9 (5.4%)	4 (5.6%)	1.000
Sex				
Female	94 (39.3%)	64 (38.3%)	30 (41.7%)	
Male	145 (60.7%)	103 (61.7%)	42 (58.3%)	0.733
Multiple_injuries				
No	180 (75.3%)	127 (76.0%)	53 (73.6%)	
Yes	59 (24.7%)	40 (24.0%)	19 (26.4%)	0.812
Craniotomy				
No	171 (71.5%)	121 (72.5%)	50 (69.4%)	
Yes	68 (28.5%)	46 (27.5%)	22 (30.6%)	0.751
Subarachnoid_hemorrhage				
No	169 (70.7%)	120 (71.9%)	49 (68.1%)	
Yes	70 (29.3%)	47 (28.1%)	23 (31.9%)	0.662
Hydrocephalus				
No	206 (86.2%)	145 (86.8%)	61 (84.7%)	
Yes	33 (13.8%)	22 (13.2%)	11 (15.3%)	0.819
Muscle_tone				
Unable to measure	78 (32.6%)	55 (32.9%)	23 (31.9%)	
Weak	37 (15.5%)	26 (15.6%)	11 (15.3%)	
Normal	77 (32.2%)	50 (29.9%)	27 (37.5%)	
High	47 (19.7%)	36 (21.6%)	11 (15.3%)	0.589
Etiology				
Traumatic brain injury	95 (39.7%)	67 (40.1%)	28 (38.9%)	
Intracerebral hemorrhage	117 (49.0%)	81 (48.5%)	36 (50.0%)	
Cerebral infarction	7 (2.9%)	5 (3.0%)	2 (2.8%)	
Others	20 (8.4%)	14 (8.4%)	6 (8.3%)	0.997
Patient’s_state_of_consciousness				
VS	150 (62.8%)	109 (65.3%)	41 (56.9%)	
MCS-	68 (28.5%)	47 (28.1%)	21 (29.2%)	
MCS+	16 (6.7%)	7 (4.2%)	9 (12.5%)	
Emergence from MCS	5 (2.1%)	4 (2.4%)	1 (1.4%)	0.111
Age				
Mean ± SD	53.7 ± 15.0	52.6 ± 16.1	56.1 ± 12.0	0.067
Hb				
Mean ± SD	101.5 ± 15.7	101.9 ± 15.8	100.7 ± 15.5	0.606
D_dimer				
Mean ± SD	3.2 ± 4.5	3.2 ± 4.5	3.4 ± 4.6	0.703
Serum_albumin				
Mean ± SD	35.8 ± 6.9	36.1 ± 7.6	35.3 ± 5.1	0.374
Length_of_hospital_stay				
Mean ± SD	31.4 ± 14.1	31.8 ± 15.0	30.4 ± 11.6	0.445
CRS-R				
Mean ± SD	7.3 ± 3.2	7.2 ± 3.1	7.6 ± 3.4	0.329
GCS				
Mean ± SD	8.2 ± 1.8	8.2 ± 1.7	8.3 ± 1.9	0.543
FOUR				
Mean ± SD	12.0 ± 2.2	11.9 ± 2.1	12.2 ± 2.4	0.305

### 3.2 Feature selection

**Figure 1:**
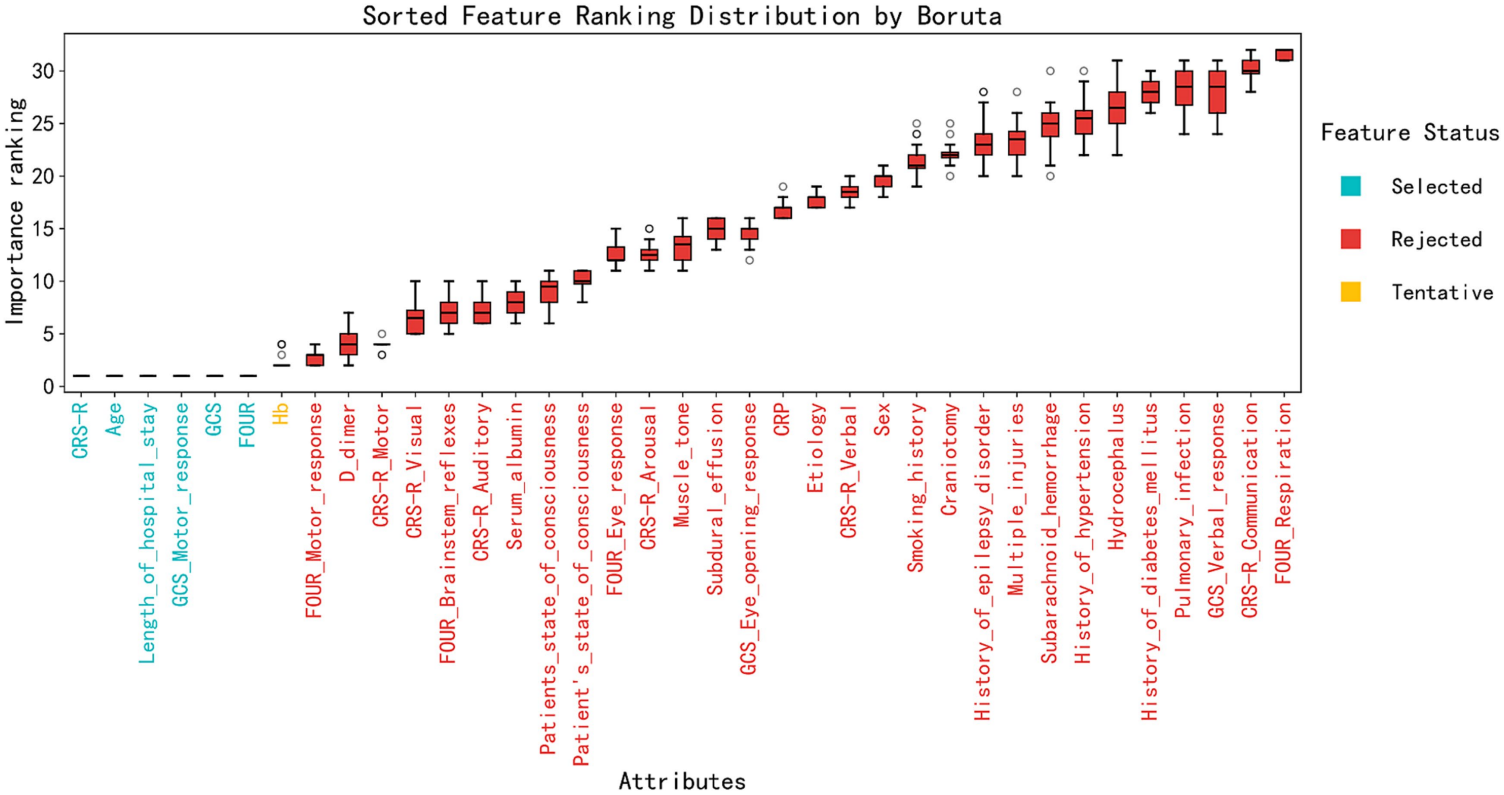
Sorted feature ranking distribution by Boruta. The feature ranking distribution was determined using the Boruta algorithm. The importance of each attribute is represented by the median importance score, with the box plots illustrating the distribution of importance scores for each feature. The attributes are sorted based on their median importance score, with the most important features on the right. The color coding indicates the feature status: selected (blue), rejected (red), and tentative (yellow).

In the feature selection process, we used the Boruta algorithm based on random forests and the Recursive Feature Elimination (RFE) algorithm to identify relevant features from an initial set of 37. The Boruta algorithm accurately identified 8 key features that played a significant role in predicting patient prognosis, including the CRS-R total score, age, length of hospitalization, GCS motor response, GCS total score, and FOUR total score. At the same time, Hb was labeled as “Tentative,” indicating that this feature may have some predictive value, but its statistical significance was somewhat weaker compared to the features that were clearly selected. The remaining features were deemed “Rejected,” suggesting that they contributed minimally to predicting patient prognosis (Figure 1).

**Figure 2:**
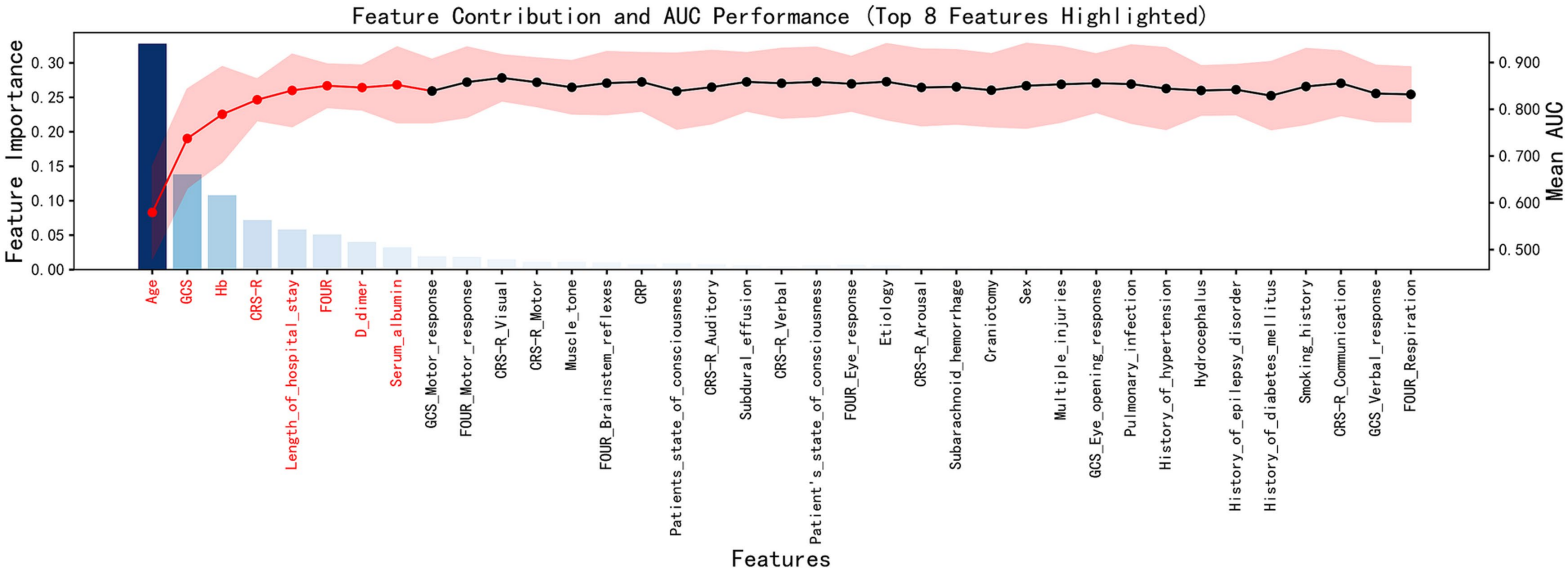
Feature Contribution and AUC Performance. The contribution of individual features to model performance, along with the AUC results, underscored the significance of the top eight most important features.

To further validate the importance of these features, we employed the Recursive Feature Elimination (RFE) algorithm. Our analysis revealed that features such as age, CRS-R, Hb, and FOUR ranked highly in terms of feature contribution, highlighting their crucial role in shaping the model’s predictive capability and marking them as key factors in predicting patient outcomes. Additionally, the model’s average AUC value gradually stabilized as more features were added, indicating that the selected feature set sufficiently supported the model’s predictive performance. Further addition of features did not significantly improve the model’s performance (Figure 2).

Throughout the feature selection process, we integrated the conclusions from both methods and carefully selected the features that made significant contributions to predicting patient outcomes. These included the CRS-R score, length of hospitalization, FOUR score, and hemoglobin level, among others. These features not only demonstrated high importance within the model but also underwent rigorous validation by the Boruta algorithm, ensuring the scientific rigor and robustness of the feature selection process. Based on this procedure, we meticulously constructed a highly efficient and accurate feature set for subsequent model development. This not only significantly enhanced the model’s predictive capability but also greatly increased its value and reliability in practical applications.

### 3.3 Multi-model ensemble analysis for classification

**Figure 3:**
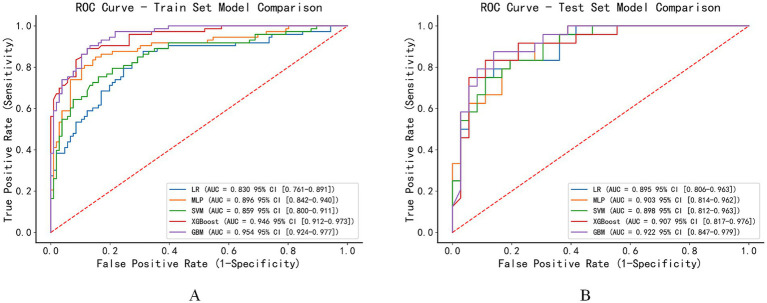
ROC curves for model performance comparison. **(A)** ROC curves of different machine learning models on the training set. **(B)** ROC curves of different machine learning models on the test set.

This study utilized five machine learning models to predict the prognosis of patients with chronic consciousness disorders, specifically LR, MLP, SVM, XGBoost, and GBM. Figure 3 displays the ROC curves for each model in the training set, with the GBM model showing the best performance (AUC = 0.954, 95% CI: 0.924–0.977). The MLP model followed, with an AUC of 0.896 (95% CI, 0.842–0.940), followed by GBM (AUC = 0.946, 95% CI: 0.912–0.973), LR (AUC = 0.830, 95% CI: 0.761–0.891), and SVM (AUC = 0.859, 95% CI: 0.800–0.911).

On the test set (Figure 3), the performance of all models slightly decreased, yet they still maintained good predictive power. The GBM model continued to perform the best (AUC = 0.922, 95% CI: 0.847–0.979), followed by XGBoost (AUC = 0.907, 95% CI: 0.817–0.976), MLP (AUC = 0.903, 95% CI: 0.814–0.962), SVM (AUC = 0.898, 95% CI: 0.812–0.963), and LR (AUC = 0.895, 95% CI: 0.806–0.963).

Compared with the LR, the GBM demonstrated a significantly higher threshold-based net reclassification improvement (Thr-NRI = 0.403, 95% CI: 0.069–0.714, *p* = 0.019), indicating improved patient risk reclassification at predefined clinical risk thresholds.

**Figure 4:**
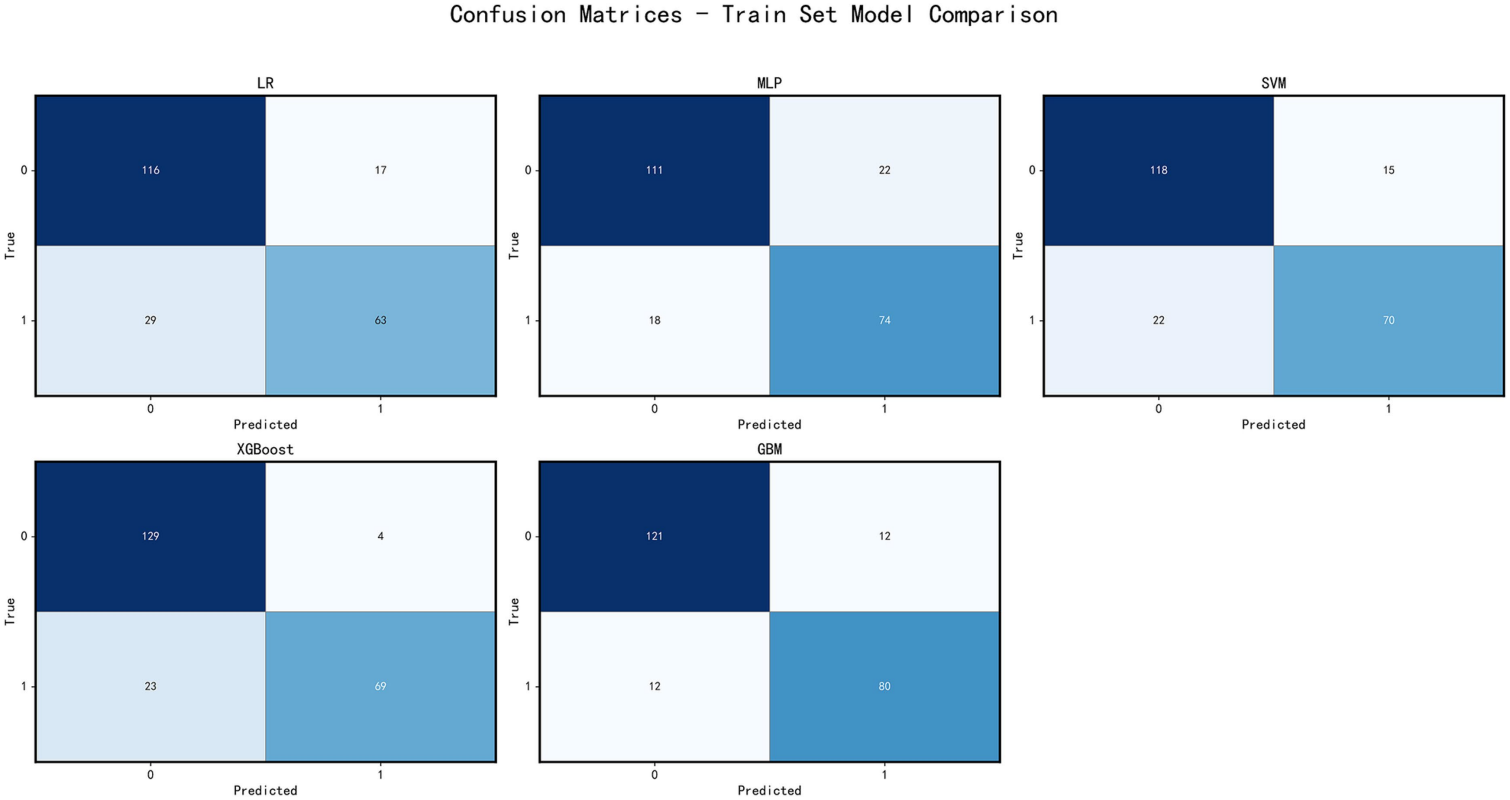
Model performance evaluation. Confusion matrices of five machine learning models (LR, MLP, SVM, XGBoost, GBM) on the training set. Each matrix shows the classification results, allowing comparison of model accuracy and classification patterns.

This study evaluated the classification performance of each model using confusion matrices on both the training and test sets. On the test set, the GBM model exhibited the highest specificity (0.977), accurately identifying patients with poor prognosis. However, its sensitivity was relatively low (0.621), resulting in the misclassification of some patients with good prognosis. The XGBoost model had a specificity of 0.930 and sensitivity of 0.690, showing a more balanced performance. The SVM and MLP models had sensitivities of 0.690 and 0.62, respectively, but their specificities were lower. The Logistic Regression model showed a sensitivity of 0.654 and specificity of 0.883, with overall lower accuracy (Figure 4).

**Figure 5:**
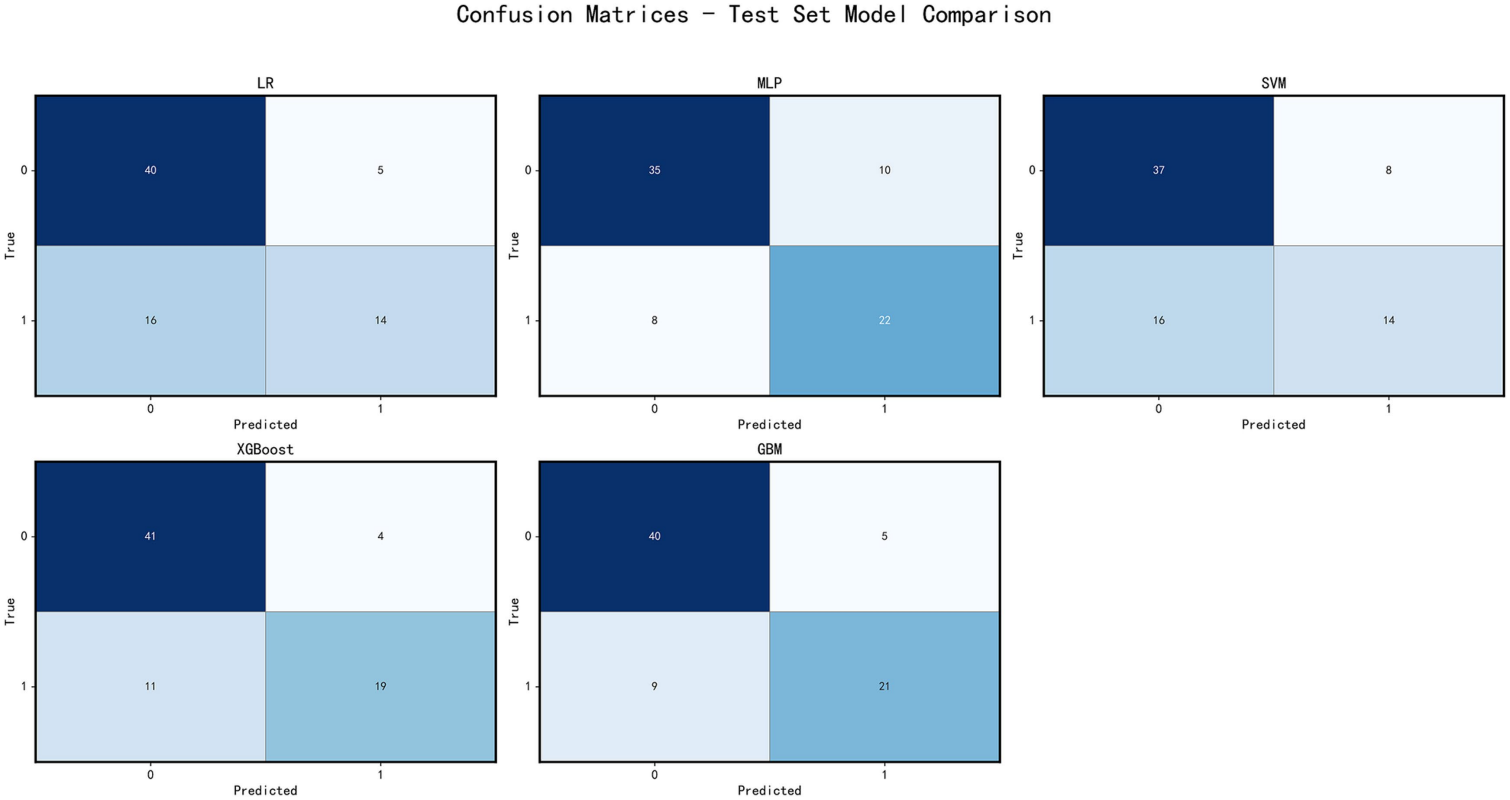
Model performance evaluation. Confusion matrices of five machine learning models on the testing set. Each matrix shows the classification results, allowing comparison of model accuracy and classification patterns.

On the training set, all models demonstrated high accuracy and low error rates. XGBoost exhibited the best sensitivity (0.750), while the GBM model excelled in specificity (0.909; Figure 5).

**Figure 6:**
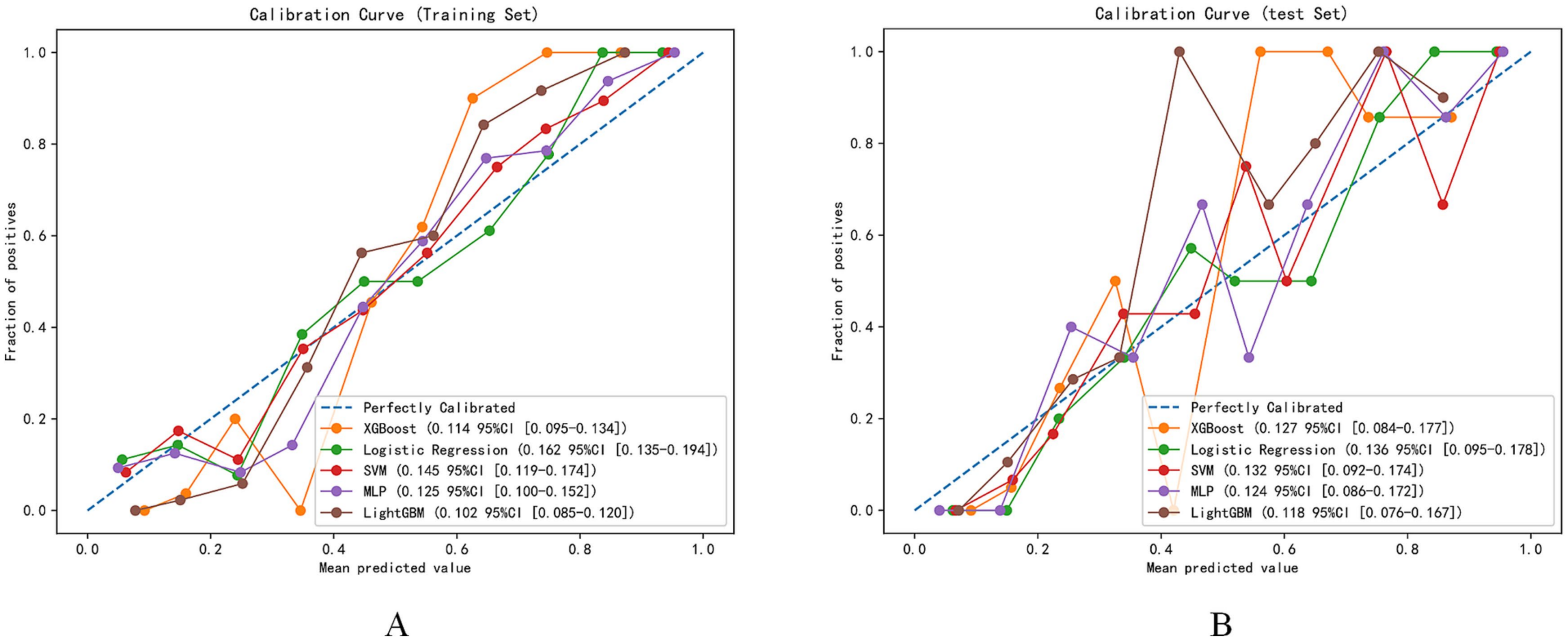
Model calibration evaluation. **(A)** Calibration curves for different machine learning models on the training set. **(B)** Calibration curves for different machine learning models on the test set.

The model calibration performance evaluation (Figure 6) revealed that the GBM model had the smallest expected calibration error on the training set. On the test set, all models showed similar calibration performance, with expected calibration errors ranging from 0.076 to 0.167.

**Figure 7:**
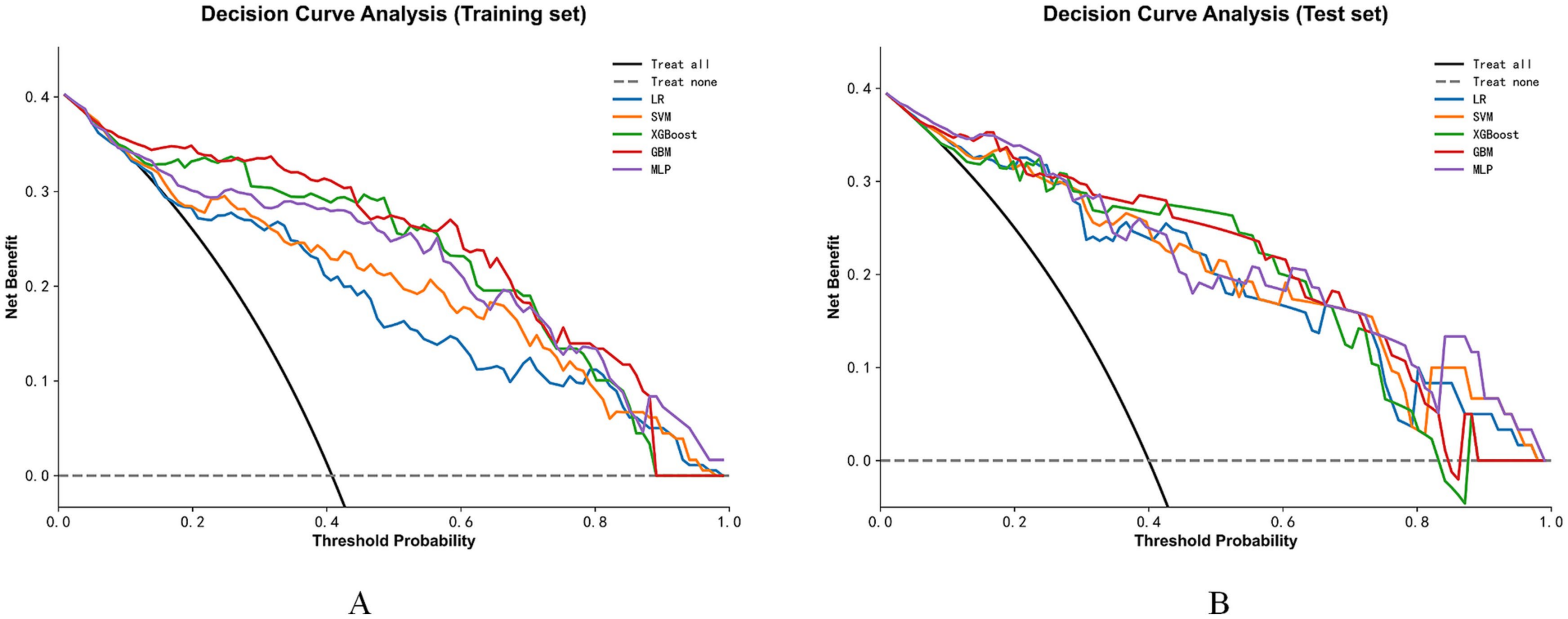
Decision curve analysis. **(A)** The net benefits of different machine learning models across varying threshold probabilities in the training set; **(B)** The corresponding performance in the test set. The black and blue dashed lines represent the reference net benefits of the “Treat all” and “Treat none” strategies, respectively.

Decision curve analysis (Figure 7) further confirmed that, across most clinically relevant decision threshold ranges, all machine learning models offered higher net benefits compared to the “treat-all” or “treat-none” strategies, with the GBM model performing best across most threshold intervals.

**Figure 8:**
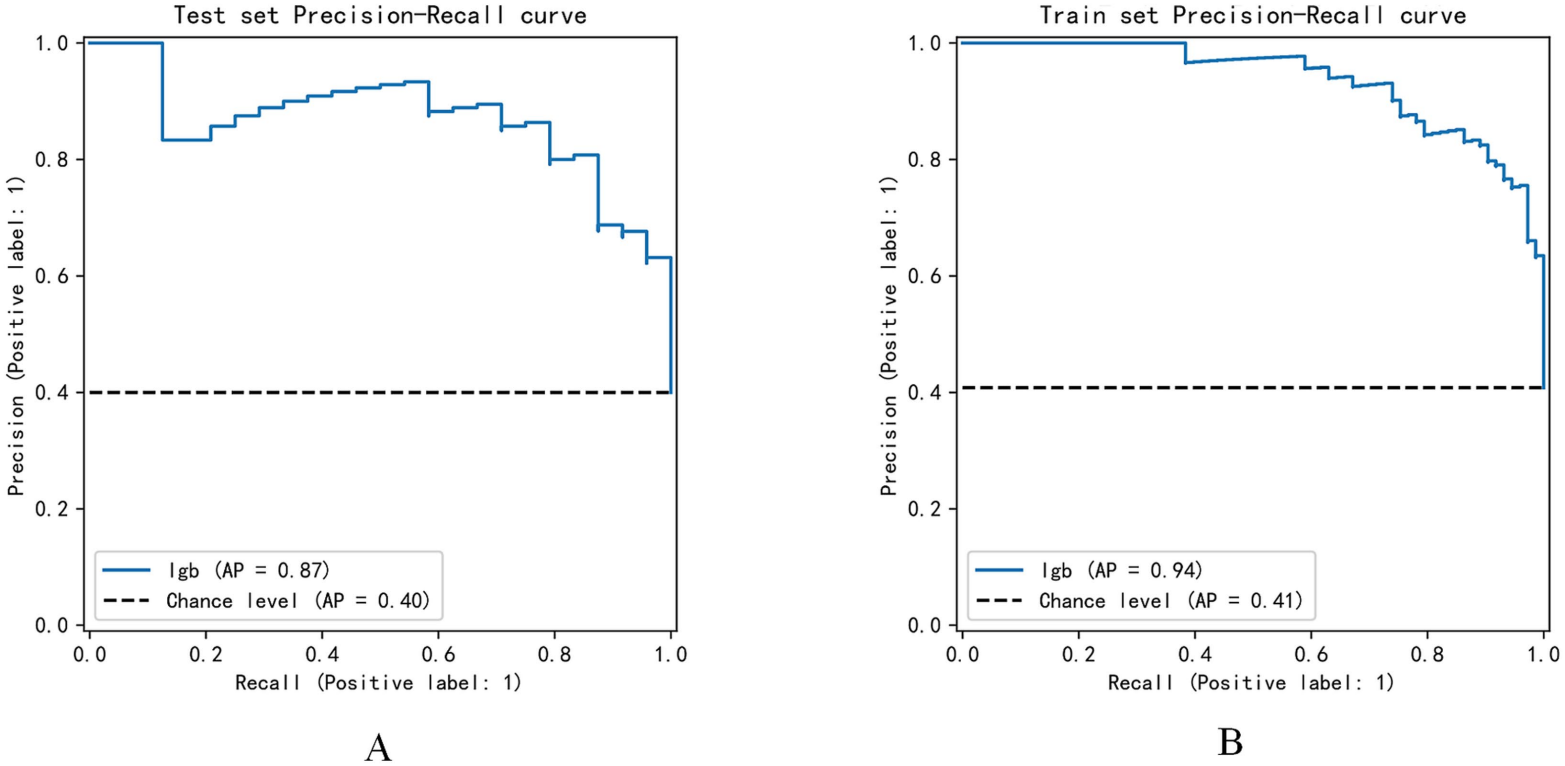
Precision-recall curve. **(A)** Precision and recall performance of the LGB model on the test set; **(B)** Performance on the training set. The x-axis represents recall, and the y-axis represents precision. The area under the curve (AP) reflects the model’s overall ability to identify positive samples, while the dashed line indicates the performance of a random classifier.

Precision-recall curve analysis (Figure 8) indicated that the GBM model achieved an average precision (AP) of 0.87 on the test set, significantly higher than the baseline level (0.40). On the training set, the average precision (AP) was 0.94, substantially higher than the baseline level (0.41), confirming that the model maintained excellent classification performance across different thresholds.

### 3.4 Model interpretability and application

Based on feature importance analysis and the SHAP value explanation framework, this study systematically identified the key factors influencing prognosis prediction in patients with chronic consciousness disorders. Through the visualization of SHAP summary plots, the impact of core features, including the CRS-R total score, age, GCS total score, and FOUR total score, was quantitatively analyzed. The SHAP feature importance plot, arranged in descending order of the absolute mean SHAP values, clearly demonstrated that these four indicators dominate the prediction model, with their cumulative contribution to the explained variance significantly higher than that of other variables. This confirmed the central role of these features in prognosis prediction.

Further analysis revealed that higher scores on the CRS-R, FOUR, and GCS scales were significantly positively correlated with favorable prognosis, highlighting the critical role of neurological function assessments in identifying recovery potential. In contrast, the age variable showed a significant negative correlation, suggesting that older age independently exerts a negative effect on prognosis. Additionally, while the weights of hospital stay duration, hemoglobin levels, and D-dimer concentrations were relatively lower, fluctuations in their SHAP values still significantly influenced the prediction outcomes, suggesting that these clinical parameters play an essential, though auxiliary, role in prognosis evaluation.

**Figure 9:**
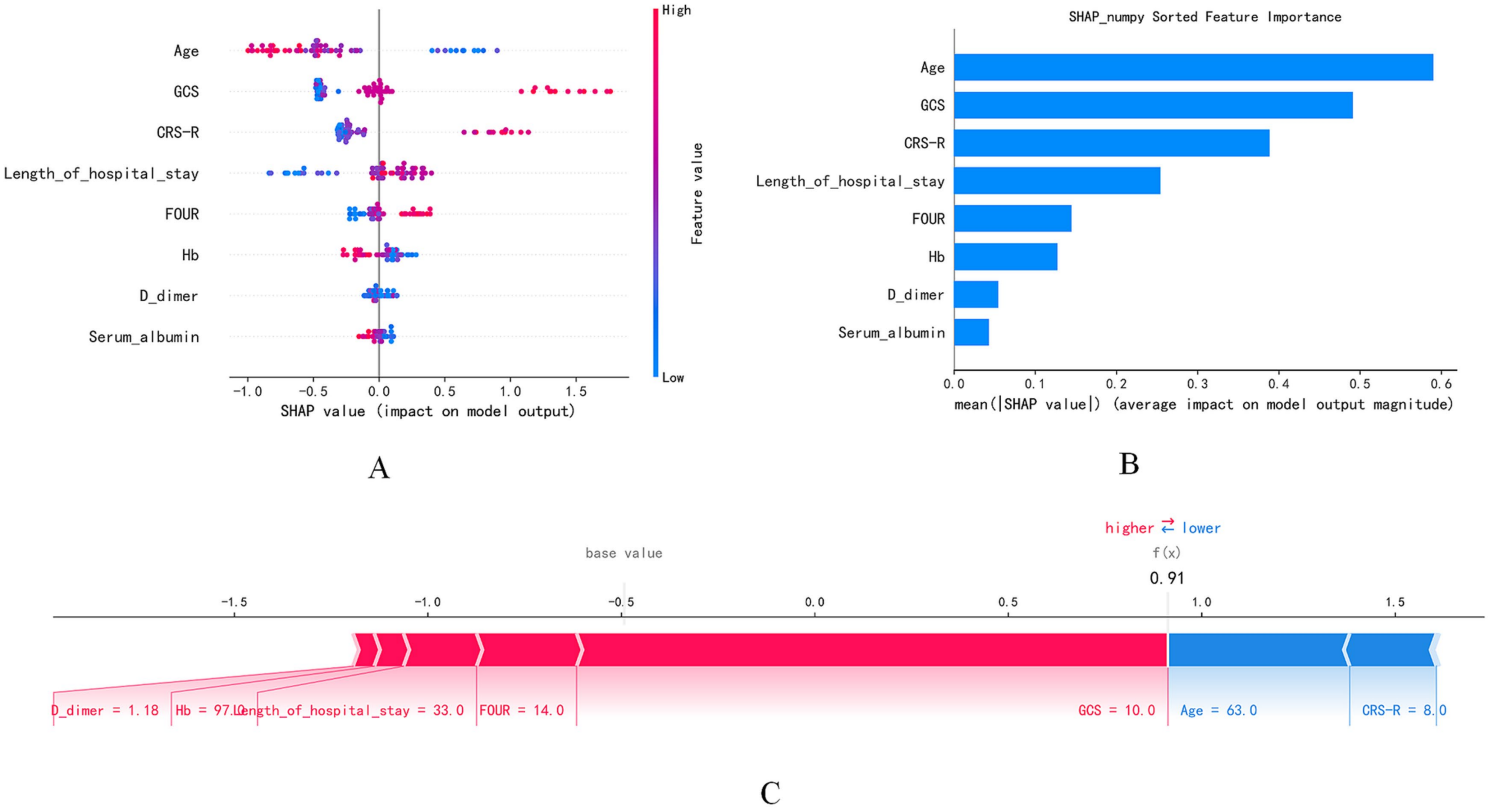
SHA *p* value analysis. **(A)** Displays the direction and magnitude of each feature’s impact on model output, with dot color indicating the feature value; **(B)** shows the mean SHAp value of each feature, reflecting its overall importance in the model; **(C)** presents the SHAP force plot of a single sample, visualizing the positive and negative contributions of individual features to the prediction, where red indicates features that increase the model output and blue indicates those that decrease it.

Case-specific analysis using SHAP force plots provided individualized insights into the model’s predictions. For example, in a typical case, features such as a FOUR score of 14.0, GCS total score of 10.0, CRS-R total score of 8.0, and a hospital stay of 33.0 days positively shifted the SHAP values, significantly increasing the likelihood of predicting a favorable prognosis. In contrast, indicators such as D-dimer concentration (1.18), hemoglobin level (97.0 g/L), age (63.0 years), and serum albumin level (32.0 g/L) contributed negative SHAP values, thereby decreasing the predicted probability of a favorable outcome. This combined approach of group feature analysis and individual case validation strengthens the statistical robustness of the model and enhances its clinical interpretability and practical utility (Figure 9).

Based on the above findings, the GBM model was selected as the optimal model. It not only demonstrated excellent predictive performance but also provides intuitive decision support for clinicians, enabling them to better understand and apply the model’s predictions, thereby optimizing treatment plans and resource allocation for patients with prolonged disorders of consciousness pDOC.

## 4 Discussion

This study evaluates an integrative machine-learning prognostic framework that combines routinely collected clinical indicators to predict functional outcome (Good vs. Poor GOS-E). By quantifying predictive performance and examining how conventional variables contribute jointly within a unified model, our findings provide evidence on the utility of data-driven integration of established clinical information for outcome stratification in this population.

The model demonstrated favorable overall discriminative performance, indicating that routinely available clinical indicators contain meaningful prognostic information when leveraged in combination. The observed operating characteristics, characterized by relatively high specificity with moderate sensitivity, suggest that the model adopts a more conservative strategy in identifying good functional outcomes. Accordingly, model outputs should be interpreted as supportive risk information, with decision thresholds selected based on the relative clinical consequences of false-positive and false-negative classifications.

The multi-model ensemble prediction model developed in this study demonstrated favorable performance in predicting the prognosis of patients with chronic disorders of consciousness. After training, testing, and validation, the GBM model showed comparatively robust predictive performance among the evaluated models. In the training set, the GBM achieved an ROC of 0.954 (95% CI, 0.924–0.977), reflecting its ability to capture complex patterns in the data. This performance may be attributed to the gradient boosting mechanism, which iteratively optimizes model performance. While LR and MLP also demonstrated reasonable predictive ability, their performance in terms of generalizability and stability appeared relatively limited. Furthermore, Thr-NRI analysis suggested that GBM outperformed LR in risk reclassification, indicating potential advantages in clinically relevant risk stratification. Moreover, DCA supported the potential clinical utility of the model across a wide range of threshold probabilities, particularly between 0.2 and 0.6.

The key prognostic prediction factors identified in this study systematically cover a multidimensional assessment framework for clinical characteristics in patients with consciousness disorders. Among these, the CRS-R, GCS, and FOUR total scores, as core indicators of neurological function assessment, play an essential role in prognosis prediction. As the most widely used behavioral assessment scale in clinical practice, the CRS-R score directly reflects the patient’s level of consciousness recovery, and its optimal value, determined through repeated assessments, can effectively enhance diagnostic reliability. A study by Xu et al. suggested that a CRS-R score ≤7 could serve as a high-risk warning indicator, significantly increasing the biological risk of progression to permanent consciousness impairment (28). The GCS scale, a classic tool for assessing the depth of coma, shows a significant correlation with consciousness state and prognosis, providing a crucial reference for evaluating the acute phase of the condition. However, the GCS is limited by its focus on three dimensions—eye, verbal, and motor responses—thereby limiting its assessment of overall neurological integrity (8). The FOUR score system expands the assessment dimensions of the GCS by integrating additional indicators such as eye movement responses, motor function, brainstem reflexes, and breathing patterns, offering a more comprehensive neurological evaluation system. From a neuroanatomical functional localization perspective, the CRS-R is more sensitive to cortical function status (29),while the FOUR scale excels at detecting signs of brainstem recovery, and the GCS focuses on evaluating motor responses of the limbs (30, 31). The combined use of these three scales forms a comprehensive assessment system that spans from cortical to brainstem function, providing a multi-layered quantitative basis for accurately charting the trajectory of neurological function recovery and designing individualized rehabilitation strategies.

Age and length of hospital stay are critical prognostic factors that significantly influence the prognosis of patients with consciousness disorders. From a neurobiological perspective, older patients experience a marked decline in cognitive reserve due to physiological neurodegenerative changes. Their synaptic remodeling and neural plasticity abilities diminish progressively, leading to a significantly reduced capacity for neurological recovery after brain injury, compared to younger individuals. In contrast, children in the brain development phase demonstrate a unique advantage in neurorepair. According to the theory of neurofunctional reorganization, children’s brains, when injured, can achieve functional recovery through compensation mechanisms in unaffected brain regions, particularly in motor function recovery, where unexpected rehabilitation effects are often seen. Recent clinical studies have provided empirical evidence supporting this conclusion. A large-scale cohort study by Duan et al. showed that the consciousness recovery rate in pediatric patients with pDoC significantly increased within 12 months post-injury: 73.1% in the TBI group and 53.9% in the non-TBI group (32). Subgroup analysis revealed that children under the age of 4, diagnosed with TBI, exhibited particularly high consciousness recovery potential, with significantly greater neurofunctional recovery efficiency than other age groups. This study suggests that age and injury type should be core components of individualized prognosis assessment systems for patients with consciousness disorders. Length of hospitalization emerged as a significant predictor; however, its interpretation warrants caution. In this study, it represents the total inpatient stay in the rehabilitation setting. This variable likely reflects a mixture of baseline functional status at rehabilitation admission, the pace of functional gains, intercurrent medical events, and discharge processes influenced by social support and institutional practice. Therefore, we interpret “length of hospitalization” primarily as an accessible marker of rehabilitation complexity and recovery trajectory rather than a causal determinant of GOS-E.

The predictive model developed in this study holds significant clinical value as a tool for early, accurate prognosis assessment. It can assist clinicians in formulating personalized treatment plans, optimizing resource allocation, and improving treatment outcomes. Additionally, it provides objective prognostic information for patients’ families, enhancing treatment adherence.

### 4.1 Limitations section

This study is limited by its single-center design, which may introduce selection bias and reduce generalizability. Although multiple routine clinical predictors were included, unmeasured factors may still influence prognosis, and the lack of neurophysiological and advanced neuroimaging data may constrain accuracy and interpretability. The model also shows high specificity but moderate sensitivity for Good vs. Poor GOS-E, potentially underestimating recovery in some patients; thus, predictions should be used cautiously with clinically appropriate thresholds. Multicenter, larger-sample validation is warranted.

## Data Availability

The raw data supporting the conclusions of this article will be made available by the authors, without undue reservation.
